# The Effects of Gestational Diabetes on Fetus: A Surveillance Study

**DOI:** 10.7759/cureus.35103

**Published:** 2023-02-17

**Authors:** Zahra Ejaz, Ayesha Azhar Khan, Syed Sebghat Ullah, Muhammad Aamir Hayat, Muhammad Arslan Maqbool, Asma Amin Baig

**Affiliations:** 1 Department of Gynecology and Obstetrics, Tentishev Satkynbai Memorial Asian Medical Institute, Kant, KGZ; 2 Department of Obstetrics and Gynecology, Arif Memorial Teaching Hospital, Rashid Latif Medical Complex, Lahore, PAK; 3 Department of General Surgery, Xinjiang Medical University, Ürümqi, CHN; 4 Department of Public Health, Tentishev Satkynbai Memorial Asian Medical Institute, Kant, KGZ; 5 Department of Medicine, Arif Memorial Teaching Hospital, Rashid Latif Medical Complex, Lahore, PAK

**Keywords:** effects of diabetes on fetus, outcomes of gdm on fetus, macrosomia, gestational diabetes mellitus, diabetes, gestation

## Abstract

Introduction: Gestational diabetes is an intolerance to glucose diagnosed during pregnancy that goes away postpartum. Gestational diabetes may result in outcomes such as birth trauma, increased rates of cesarean sections, and macrosomia. This study aims to determine the outcomes of gestational diabetes mellitus (GDM) on maternal and fetal health in a tertiary care hospital setting.

Materials and methods: This is a retrospective study of 52 patients who presented with gestational diabetes mellitus (GDM) and were treated at Tentishev Satkynbai Memorial Asian Medical Institute, Kyrgyzstan, between April 2021 and January 2022. The information was taken from the medical records of the patients. The baby's age, the mother's body mass index (BMI), history of pregnancy, deaths, birth weight, and the number of births were all taken into account.

Results: Out of all the cases during the study period at the Tentishev Satkynbai Memorial Asian Medical Institute, Kyrgyzstan, 52 were found to be complicated with gestational diabetes mellitus, which is 2.7% of the total deliveries. There was a significant difference found among both study groups in gestational age and history of GDM. The neonatal intensive care unit (NICU) admission rate of neonates born to GDM mothers was found to be significant with a difference of 10.9% (p < 0.0003), which is higher compared to the control group.

Conclusion: Incidences of macrosomia, NICU admissions of preterm babies, and large for gestational age (LGA) and increased rates of hypertensive disorders were found among GDM pregnancies compared to control cases. The study shows higher rates of maternal and fetal/neonatal complications in females with GDM.

## Introduction

Gestational diabetes mellitus (GDM) is an intolerance to glucose that is diagnosed during pregnancy [1]. This disorder commonly fades away postpartum; also, in a few cases, this sustains even after the pregnancy [2]. If their blood glucose levels are well controlled, females with GDM disorder usually have healthy neonatal births. These results can be achieved with either diet or insulin, combined with maintaining an appropriate body weight [3]. Gestational diabetes cases can result in negatively impacting pregnancy outcomes such as birth trauma, increased rates of cesarean sections (10% absolute risk), and macrosomia (14% absolute risk), and the outcome measurements may differ depending on variables such as screening methods, population cohorts, and diagnostic criteria [4].

The management of GDM is based on establishing control over the serum glucose levels in GDM patients by measuring glucose levels repeatedly both during home monitoring and via glycosylated hemoglobin [5]. A large population affected by GDM usually responds to a controlled diet via diet therapy alone, but the remaining subjects need insulin in addition to the dietary changes [6]. Controlling the blood sugar levels of patients affected by GDM via lifestyle and diet has shown improvement in perinatal outcomes in 70%-85% of patients in a recent study [7].

This study aims to determine the outcomes of phototherapy, jaundice, macrosomia birth, and other factors related to GDM on both mothers and fetuses/neonates in a Kyrgyz population and to compare them with nondiabetic pregnancy outcomes in a tertiary care hospital setting.

## Materials and methods

This is a retrospective study of 52 patients presenting with GDM and treated at the Tentishev Satkynbai Memorial Asian Medical Institute, Kyrgyzstan, between April 2021 and January 2022. The ethical committee of the Tentishev Satkynbai Memorial Asian Medical Institute, Kyrgyzstan, approved the undertaking of the study, and the data used in the current study were obtained from the medical records of the patients, where the age, body mass index (BMI), pregnancy complications, mortality, and birth weight data of the neonate and parity were collected and processed for this study.

The control group was selected using hospital databases, as they had not presented with GDM at any stage of their pregnancy, and the age, parity, and BMI of the control group were matched with those of the GDM group in the study. All the subjects selected for the study were hospitalized and delivered to the hospital's gynecology department. The measurements taken to record neonatal outcomes were weight at the time of birth, hypoglycemia, respiratory distress syndrome (RDS), hypocalcemia, bilirubin, and intensive care unit (ICU) admissions. The Apgar scores used in the study were collected from the hospital records at one, five, and 10 minutes postpartum.

The screening for GDM was done during the study period using a selective screening method that is based on factors such as prepregnancy GDM, age of the mother, weight, family history of diabetes, and macrosomia birth history. During the gestational period, the screening took place between weeks 20 and 25. If the results obtained via venipuncture using the glucose oxidase method exceeded 140 mg/dL, the patient would then go through another test in which a 100 g glucose oral test was administered, and after three hours, the following cutoff values were used: fasting blood sugar of 100 mg/dL; after one hour, 190 mg/dL; after two hours, 165 mg/dL; and after three hours, 165 mg/dL [3]. Patients who were diagnosed with GDM have been prescribed a diet within the 1800 kcal diabetic diet for one week, followed by a fasting blood sugar test if the results showed a fasting blood sugar value of less than 100 mg/dL and a postprandial value of less than 125 mg/dL; those patients were managed by adhering to a specialized diet only. Patients presenting with results higher than these cutoff values were prescribed insulin to control their blood glucose levels. The number of GDM patients treated only with dietary changes was 40; the remaining 12 patients required insulin on top of diet changes. The patients included in the study were scheduled for follow-up every two weeks after GDM was confirmed. Labor was induced after 40 weeks in patients who had been treated for GDM solely with diet. Some patients required an earlier labor induction due to toxemia, depending on their biophysical profile. The blood sugars of neonates born to diabetic mothers were measured after delivery and continued to be measured until the values were stable. Intravenous glucose was administered to babies with hypoglycemia, and feeding was initiated as soon as possible. Blood sugar measurements on neonates in the control group were taken only when indicated.

Statistical Package for Social Sciences (SPSS) version 23 (IBM SPSS Statistics, Armonk, NY) was used to perform statistical analysis, and a chi-square test and Fisher's exact test were used to assess statistical significance. Confidence intervals of 90% and odds ratios (OR) were calculated. A p-value of 0.05 was considered significant. To estimate the confidence interval and odds ratio, a multivariate logistic regression was employed. The results are shown as mean standard deviation, "n" represents frequency, and nominal data are presented as a percentage (%).

## Results

Out of all the cases during the study period at the Tentishev Satkynbai Memorial Asian Medical Institute, Kyrgyzstan, 52 were found to be complicated with gestational diabetes mellitus, which is 2.7% of the total deliveries. The demography of the females affected with GDM can be viewed in Table 1.

**Table 1:** Maternal outcomes GDM, gestational diabetes mellitus; BMI, body mass index; DM, diabetes mellitus; OR, odds ratio; CI, confidence interval *Statistically significant

Characteristics	GDM, N = 52 (%)	Control, N = 52 (%)	P-value	OR (95% CI)	Adjusted OR (95% CI)
Age (in years)	28 ± 7.5	29.2 ± 6.8	0.3947	-	-
Parity: 0, 1, and ≥2	9 (16.9%), 15 (29.3%), and 28 (53.8%)	8 (15.3%), 18 (34.6%), and 26 (50.1%)	-	-	-
Delivery time (weeks) mean age for gestation	38.5 ± 1.4	39.4 ± 1.6	0.0001*	(0.082-0.518)	(0.101-0.645)
BMI (kg/m^2^)	28.4 ± 1.5	27.1 ± 1.6	0.1490	-	-
DM family history	22 (41.43%)	17 (32.34%)	0.060	-	-
Prior history of (H/O) GDM	10 (19.5%)	4 (7.7%)	0.0004*	2.901 (1.597-5.268)	2.072 (1.064-4.745)
Previous H/O macrosomia	4 (7.3%)	2 (4.5%)	0.3121	-	-
Previous stillbirth	1 (1.4%)	1 (1.8%)	1.000	-	-
Hypertension	9 (18.2%)	3 (5.9%)	<0.0001*	3.538 (1.834-6.824)	2.958 (1.251-6.313)
Preterm delivery	6 (11.42%)	3 (5.11%)	0.0233	2.434 (1.167-5.082)	2.013 (1.059-4.862)
C-section	12 (24.11%)	6 (12.33%)	0.0018	2.269 (1.364-3.769)	2.134 (1.123-2.934)
Polyhydramnios	2 (3.22%)	1 (1.44%)	0.337	-	-
Oligohydramnios	1 (2.71%)	0 (0.91%)	0.2845	-	-
Labor induction	17 (31.82%)	6 (12.31%)	<0.0001*	3.334 (2.037-5.458)	2.702 (1.852-5.224)

Both study groups showed a significant difference in gestational age and history. On multivariate logistic regression high-risk variable, the values found are as follows: Hypertensive disorders were found to be p < 0.0001, the p-value was 0.001 for the induction of labor, and the value of preterm delivery was p < 0.236. The multivariate analysis showed that females who were previously diagnosed with GDM were at a higher risk of developing GDM in future pregnancies.

Babies born to GDM females had a higher mean birth weight (macrosomia) compared to control cases' mean birth weight, which is elaborated in Table 2.

**Table 2:** Neonatal outcome values GDM, gestational diabetes mellitus; RDS, respiratory distress syndrome; ICU, intensive care unit; N/A, not significant/applicable *Statistically significant

Outcome	GDM, N = 52 (%)	Control, N = 52 (%)	P-value	Odds ratio (95% confidence interval)	Odds ratio (adjusted) (95% confidence interval)
Mean birth weight (grams)	3544 ± 466	3357 ± 332	<0.0001	(113.18-264.82)	(105-231.40)
Large for gestational age	8 (14.54%)	3 (5.11%)	0.0012	3.233 (1.586-6.395)	3.342 (1.465-6.376)
Macrosomia birth	7 (12.71%)	3 (5.1%)	0.0185	2.76 (1.343-5.618)	2.68 (1.233-5.494)
Small for gestational age	4 (7.4%)	3 (6.87%)	1.01	N/A	N/A
Birth weight of <2.500 kg	2 (3.68%)	2 (3.21%)	1.0	N/A	N/A
ICU entry	9 (16.4%)	3 (5.5%)	0.0003*	3.391 (1.713-6.712)	2.954 (1.732-6.805)
RDS	1 (1.4%)	0 (0.9%)	0.6233	N/A	N/A
Hypoglycemia	1 (2.74%)	0 (0.91%)	0.2789	N/A	N/A
Incidence of jaundice in newborns	4 (8.2%)	2 (4.5%)	0.1707	N/A	N/A
Phototherapy	3 (5.1%)	1 (2.7%)	0.2017	N/A	N/A
Apgar score of <7 at five minutes	2 (3.2%)	1 (2.3%)	0.543	N/A	N/A
Congenital anomalies in neonates	1 (1.4%)	1 (1.8%)	1.000	N/A	N/A
Perinatal deaths	0 13.6	0 9.1	1.0000	N/A	N/A

The neonatal intensive care unit (NICU) admission rate of neonates born to GDM mothers was 10.9%, which is significantly higher than the control group (p < 0.0003). No perinatal death was recorded in the study duration.

## Discussion

Gestational diabetes mellitus is a clinical complication diagnosed during pregnancy that is associated with a person having up to a 60% chance of developing type 2 diabetes mellitus in the later stages of life [8]. GDM, if left untreated during pregnancy, is strongly related to a higher risk of developing maternal and perinatal risks that are responsible for morbidity and mortality in some cases [9]. Patients are evaluated and screened for GDM to prevent perinatal morbidity, stillbirth, and large for gestational age (LGA) babies, which are the most common complications to avoid [10]. According to one study, excessive fetal growth has an indirect impact, with risk factors such as the patient's parity, ethnicity, maternal age, and maternal obesity being significant factors [11]. The results of this study largely conform with the results of previous studies; however, there are a few differences in outcomes. This study was conducted under the assumption that GDM patients are more likely to experience complications during pregnancy, as well as adverse effects on fetal outcomes. The results show a higher incidence of obstetric complications, including but not limited to preterm labor, preeclampsia, LGA, macrosomia, and a higher need for cesarean section, in GDM-affected females compared to the control group; the results are similar to another GDM risk score study [12].

The rate of the induction of labor was found higher in GDM cases in a recent study, which is similar to the results of our study where, in GDM cases, the rate was 31.8% [13]. The rate of cesarean section was found to almost double in the GDM group as compared to the control group in our study, which is consistent with a review study [14]. Previous cesarean section history, hypertension, macrosomia, and insubstantial fetal heart tracing were the main indicators of cesarean section in GDM-affected subjects. This study confirms the findings of a previous study that changes in diet and GDM treatment through insulin, if required, drastically change the serious perinatal morbidity rate of patients and common birth issues of neonates [15]. The GDM group included in this study showed significant rates of neonatal intensive care unit (NICU) admissions (16.4%, p < 0.0003) compared to the control group, and the rate of admission was found to be slightly higher in the GDM group that was treated with insulin. Significant differences were not found in small for gestational age (SGA) neonates and neonatal hypoglycemia and phototherapy among both groups. However, due to pregnancy risk factors and fetal complications, neonates of GDM mothers spent more time in the NICU after admission than the control group; this is due to the hospital policy of keeping GDM neonates under observation for 24 hours. In our study, the rate of NICU admissions was comparable to a previous study done in Australia [16].

An Iranian study discovered that even minor changes in blood glucose levels can result in macrosomia (abnormal fetal growth) and other complications, which can be avoided by implementing simple measures such as a controlled diet and insulin use during the gestational period [17]. The standard treatment for GDM management is diet control and insulin therapy. Oral hypoglycemic agents have also shown promise in controlling blood glucose levels during GDM without having a negative impact on fetal outcomes [18].

The study's limitations include the small sample size and the lack of evaluation of numerous biomarkers. There needs be more research with a larger sample size to assess the connections between maternal glucose levels and perinatal outcomes.

## Conclusions

In this study, the incidence of macrosomia, preterm baby NICU admissions, and LGA, and a higher incidence of hypertensive issues were found in GDM pregnancies compared to the non-GDM control group. The results of this study support the previous findings of higher rates of maternal and neonatal complications in females affected by GDM. The study concludes that tight control of GDM during pregnancy can turn out to be the one major variable that can significantly reduce the complications correlated with GDM. The study concludes that GDM is directly related to obesity and recommends that all patients be screened for GDM because even mild diabetes can have a significant impact on fetuses and pregnant females' outcomes. The study found weight as a major variable that can be altered to get positive results for both maternal and fetal outcomes.
